# NPM-ALK mediates phosphorylation of MSH2 at tyrosine 238, creating a functional deficiency in MSH2 and the loss of mismatch repair

**DOI:** 10.1038/bcj.2015.35

**Published:** 2015-05-15

**Authors:** K M Bone, P Wang, F Wu, C Wu, L Li, J T Bacani, S E Andrew, R Lai

**Affiliations:** 1 Department of Laboratory Medicine and Pathology, University of Alberta, Edmonton, Alberta, Canada; 2 Department of Chemistry, University of Alberta, Edmonton, Alberta, Canada; 3 Department of Medical Genetics, University of Alberta, Edmonton, Alberta, Canada; 4 Department of Oncology, University of Alberta, Edmonton, Alberta, Canada; 5 DynaLIFE_DX_ Medical Laboratories, Edmonton, Alberta, Canada

## Abstract

The vast majority of anaplastic lymphoma kinase-positive anaplastic large cell lymphoma (ALK+ALCL) tumors express the characteristic oncogenic fusion protein NPM-ALK, which mediates tumorigenesis by exerting its constitutive tyrosine kinase activity on various substrates. We recently identified MSH2, a protein central to DNA mismatch repair (MMR), as a novel binding partner and phosphorylation substrate of NPM-ALK. Here, using liquid chromatography–mass spectrometry, we report for the first time that MSH2 is phosphorylated by NPM-ALK at a specific residue, tyrosine 238. Using GP293 cells transfected with NPM-ALK, we confirmed that the MSH2^Y238F^ mutant is not tyrosine phosphorylated. Furthermore, transfection of MSH2^Y238F^ into these cells substantially decreased the tyrosine phosphorylation of endogenous MSH2. Importantly, gene transfection of MSH2^Y238F^ abrogated the binding of NPM-ALK with endogenous MSH2, re-established the dimerization of MSH2:MSH6 and restored the sensitivity to DNA mismatch-inducing drugs, indicative of MMR return. Parallel findings were observed in two ALK+ALCL cell lines, Karpas 299 and SUP-M2. In addition, we found that enforced expression of MSH2^Y238F^ into ALK+ALCL cells alone was sufficient to induce spontaneous apoptosis. In conclusion, our findings have identified NPM-ALK-induced phosphorylation of MSH2 at Y238 as a crucial event in suppressing MMR. Our studies have provided novel insights into the mechanism by which oncogenic tyrosine kinases disrupt MMR.

## Introduction

Anaplastic lymphoma kinase-positive anaplastic large cell lymphoma (ALK+ALCL) is a specific type of T/null-cell non-Hodgkin's lymphoma recognized by the World Health Organization Classification Scheme for hematologic malignancies.^1^ Most of these tumors are characterized by the expression of the oncogenic fusion protein NPM-ALK, which results from the reciprocal translocation *t(2;5)(p23;q35)* involving the anaplastic lymphoma kinase (ALK) and nucleophosmin (NPM) genes.^2^ Previous studies have demonstrated that NPM-ALK mediates tumorigenesis by constitutively activating various signaling pathways leading to cell cycle deregulation and enhanced survival.^3^

We previously reported that MutS homolog 2 (MSH2), a protein central to DNA mismatch repair (MMR), is tyrosine phosphorylated by NPM-ALK, leading to the disruption of MMR.^4^ MSH2 and other MMR proteins are highly expressed in normal cells, and their key function is to maintain genomic stability by correcting DNA damage and replication errors from endogenous and exogenous sources.^5^ They do so by binding and correcting single-base mismatches and insertion-deletion loops, which occur when the replication machinery 'stutters' on highly repetitive microsatellite sequences.^6^ The predominant MMR protein heterodimer is composed of MSH2:MSH6 (MutSα), and it repairs single-base mismatches, small insertion–deletion loops and DNA-damage adducts.^5^ The importance of MMR in tumor biology has been highlighted in Lynch syndrome, which is caused by hereditary germline mutations in MMR genes.^7^ Loss of MMR function and the resultant microsatellite instability (MSI) have been associated with the development and progression of a number of hematological malignancies, including acute and chronic myeloid leukemia, myelodysplastic syndrome and non-Hodgkin's lymphoma.^8, 9, 10, 11^ In our previous study, we found evidence of MSI in six out of nine ALK+ALCL tumors.^4^ MMR dysfunction as a result of progressive loss of MMR genes is also a frequent event in a variety of sporadic cancers, in which MMR-deficient cells display a significantly higher mutation rate and resistance to some chemotherapeutic agents.^12^

Post-translational modification of MMR proteins has not been extensively characterized,^13, 14, 15^ and our group described tyrosine phosphorylation (p-Y) of MSH2 for the first time in 2011.^4^ Specifically, we found evidence that NPM-ALK mediates p-Y of MSH2, deregulating MMR. In this study, we sought to further delineate this process and explore the biological significance of MSH2 p-Y. We first determined the specific tyrosine residue(s) on MSH2 that are phosphorylated by NPM-ALK by mass spectrometry. Using a specific MSH2 mutant in which NPM-ALK-mediated p-Y of MSH2 is largely abrogated, we assessed whether this newly described phosphorylation event underlies the NPM-ALK-induced MMR deficiency.

## Materials and methods

### Cell lines, cell culture and gene transfection

GP293 cells were maintained as previously described.^16^ Tet-on HEK293/NPM-ALK cells and ALK+ALCL cell Karpas 299 and SUP-M2 were cultured as previously described.^4^ Tet-on HEK293 cells were purchased from Clontech Laboratories (Mountainview, CA, USA) and Karpas 299 and SUP-M2 cells were purchased from the ATCC (Manassas, VA, USA) and were recently tested for mycoplasma infection. GP293 cells were transiently transfected with expression vectors using Lipofectamine 2000 (Invitrogen, Life Technologies, Grand Island, NY, USA) according to the manufacturer's protocol.

### Gene expression vectors and site-directed mutagenesis

His-biotin (HB)-tagged MSH2 was constructed by cloning MSH2 from pcDNA3-MSH2^17^ (a gift from Dr Meuth) into the HB-tagged vector described previously.^18^ Site-directed mutagenesis of tyrosine (Y) 238 of MSH2 to phenylalanine (F; MSH2^Y238F^) was performed using the QuikChange Site Directed Mutagenesis Kit (Agilent Technologies, Mississauga, ON, Canada). The NPM-ALK expression vector was a gift from Dr Morris.^16^

### Tandem affinity purification under denaturing conditions, on-bead protein digestion and liquid chromatography–mass spectrometry

Tandem affinity purification and liquid chromatography–mass spectrometry under denaturing conditions using GP293 cells transfected with HB-MSH2 and NPM-ALK were performed as previously described. Mass spectrometry results were confirmed using a biological replicate.^18, 19^

### Generation of Tet-on ALK+ALCL MSH2/MSH2^Y238F^ cell lines

Tet-on ALK+ALCL MSH2^Y238F^ cell lines were generated as outlined in the Supplementary Methods.

### Immunoprecipitation, His-based protein purification and western blotting

All coimmunoprecipitation and immunoprecipitation experiments were performed as previously described using 1000 μg of lysate in Cell Lytic M (Sigma Aldrich, Oakville, Ontario, Canada).^4^ No-antibody controls were included, although not always shown. Biotin-based protein purification was performed as previously described using 500 μg of lysate in RIPA buffer.^18^ These experiments were repeated at least three times. The following antibodies were used: anti-MSH2 (EMD, Billerica, MA, USA, IP/co-IP, catalog number NA27), anti-MSH6 (BD Biosciences, San Jose, CA, USA, catalog number 610919), anti-MSH2, anti-phospho-tyrosine, anti-ALK, anti-total caspase 3 (Cell Signaling Technologies, Danvers, MA, USA, catalog numbers 2017, 9416, 3633 and 14220, respectively) and anti-β-actin (Santa Cruz Biotechnology, Dallas, TX, USA; sc-47778).

### Functional assay for MMR: β-galactosidase reporter plasmid

Tet-on HEK293/NPM-ALK cells were transfected with the pCAR-OF *β-galactosidase* reporter plasmid,^20^ treated with doxycycline (DOX, Sigma Aldrich) and analyzed for β*-*galactosidase production, as previously described.^4^ Samples were read in sextuplicate, and the experiment was repeated three times.

### Functional assay for MMR: sensitivity to DNA-damage-inducing drugs

GP293 cells were transfected with HB-EV, or HB-MSH2^Y238F^ and NPM-ALK, for 24 h, and then they were plated into 48-well plates. Twenty-four hours later, cells were treated with 0, 0.5, 1, 2, 3 or 4 mM *N*-methyl-*N*-nitrosourea (MNU) for 48 h, and cell viability was assessed by MTS (Promega, Madison, WI, USA). Tet-on SUP-M2 MSH2^Y238F^ cells were treated with 0 or 500 ng/ml DOX for 48 h, followed by the addition of 0 or 100 μM MNU or 5 μg/ml 5-fluorouracil (5-FU). After 24 h, cell viability was assessed, and then re-measured every 2 days. Samples were analyzed in triplicate and the experiment was repeated three times.

### Apoptosis analysis

Apoptosis analysis was performed on Tet-on SUP-M2 and Karpas 299 MSH2^Y238F^ cells treated with 0, 100 and 500 ng/ml DOX, and stained after 48 h with Annexin-V-FITC/PI (BD Biosciences) according to the suggested protocol. Analysis was performed on a BD FACS Calibur (Flow Cytometry Laboratory, Department of Experimental Oncology, Cross Cancer Institute). Cell cycle analysis was performed using propidium iodide staining followed by flow cytometry, as previously described^21^ on Tet-on SUP-M2 MSH2^Y238F^ cells treated with 0 and 500 ng/ml DOX for 48 and 120 h, respectively. Samples were analyzed in triplicate, and the experiment was repeated three times.

### Statistical analysis

Data are expressed as mean±s.e.m., and significance was determined from three independent experiments by Student's *t*-test (GraphPad Prism, La Jolla, CA, USA). Sample size was chosen to give adequate power to the statistical calculations. No sample results were excluded from calculations.

## Results

### Identification of Y238 of MSH2 as the crucial site for NPM-ALK-induced p-Y

Using NetPhos 2.0,^22^ we analyzed the MSH2 protein sequence for potential p-Y sites that may be targeted by NPM-ALK. We identified 13 putative sites in the MSH2 protein (Supplementary Figure 1 and Supplementary Table 1). The tyrosine (Y) residue with the highest score was Y238 (0.981, * in Supplementary Figure 1), located in the connector domain required for the formation of the MMR ternary complex.

To identify the specific residue(s) phosphorylated in the presence of NPM-ALK, tandem-affinity purification was performed. To achieve this, we used GP293 cells transiently transfected with both HB-tagged MSH2 and NPM-ALK. Using these cell lysates, we performed liquid chromatography coupled with tandem-affinity purification mass spectrometry. With the total peptide sequence coverage of MSH2 being 78%, the only detectable phosphorylated residue in the presence of NPM-ALK was Y238 (Supplementary Figure 2). The same experiment was done on GP293 lysate expressing HB-MSH2 and pcDNA3, and no evidence of phosphorylation was detected.

### NPM-ALK mediates p-Y of MSH2 at tyrosine 238

**Figure 1:**
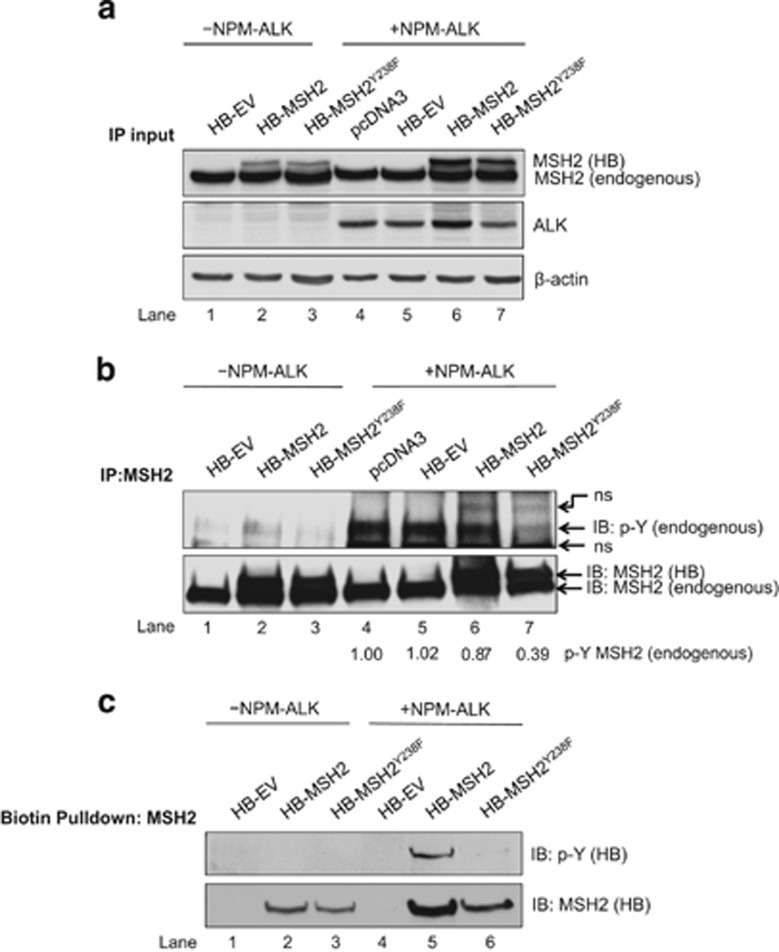
NPM-ALK mediates p-Y of MSH2 at Y238. (**a**) Western blot analysis of cell lysate from GP293 cells transfected with HB-EV (empty vector, lanes 1 and 5; negative control for HB-MSH2/MSH2^Y238F^), HB-MSH2 (lanes 2 and 6), HB-MSH2^Y238F^ (lanes 3 and 7) and pcDNA3 (lane 4), with NPM-ALK (lanes 4–7) or pcDNA3 (lanes 1–3; negative control for NPM-ALK). (**b**) IP analysis of lysate from (**a**) showing p-Y of endogenous and HB-tagged MSH2 with pcDNA3 (lanes 1–3) or NPM-ALK (lanes 4–7) expression. The blot was probed with an MSH2 antibody to confirm immunoprecipitation. Densitometry was calculated on the basis of the intensity of endogenous p-Y (upper panel) normalized to total MSH2 (lower panel) in samples with NPM-ALK expression only (lanes 4–7). (**c**) Lysate from (**a**) subjected to biotin purification using streptavidin agarose resin to detect p-Y of precipitated HB-tagged MSH2 proteins in the presence of pcDNA3 (lanes 1–3) or NPM-ALK (lanes 3–6). An MSH2 antibody was used to confirm biotin pull-down of exogenous MSH2 proteins. Results shown are representative of three independent experiments. ns, nonspecific band.

To examine the functional significance of phosphorylation of MSH2^Y238^, site-directed mutagenesis was performed on the HB-MSH2 plasmid to change the residue to F, a residue biochemically similar to Y that cannot be phosphorylated. HB-empty vector (HB-EV), HB-MSH2 or HB-MSH2^Y238F^ was transfected into GP293 cells with or without NPM-ALK expression (Figure 1a). Immunoprecipitation using an anti-MSH2 monoclonal antibody was then performed on lysate depicted in Figure 1a. In the absence of NPM-ALK (Figure 1b, lanes 1–3), there was no convincing evidence of tyrosine phosphorylation of endogenous MSH2, HB-MSH2 or HB-MSH2^Y238F^, compared with the EV control. In the presence of NPM-ALK (Figure 1b, lanes 4–7), phosphorylation of endogenous MSH2 was readily detectable. Importantly, the phosphorylation of endogenous MSH2 in the presence of HB-MSH2^Y238F^ was substantially (>60%) reduced (lane 7), suggesting that the MSH2^Y238F^ mutant exerts a dominant negative effect on endogenous MSH2 p-Y.

Rather surprisingly, phosphorylation of the HB-tagged MSH2 proteins was not detectable in this experiment. However, as shown in Figure 1c, when streptavidin agarose resin was used to pull down the exogenous HB-MSH2 or HB-MSH2^Y238F^, NPM-ALK mediated p-Y of HB-MSH2 (lane 5), whereas there was only a barely detectable signal for HB-MSH2^Y238F^ (lane 6). The discrepancy between Figures 1b and 1c regarding the phosphorylation of HB-MSH2 is likely owing to the fact that the anti-phospho-tyrosine antibody does not recognize its epitope on HB-MSH2 when it was pulled down by an anti-MSH2 antibody.

Taken together, these results suggest that NPM-ALK phosphorylated at MSH2^Y238^. Furthermore, as site-directed mutagenesis of Y238 almost completely abrogated the p-Y signal induced by NPM-ALK, Y238 is the predominant p-Y site on MSH2 in this context. In conclusion, MSH2^Y238F^ appears to have a dominant negative effect on NPM-ALK-mediated phosphorylation of endogenous MSH2.

### Enforced expression of HB-MSH2^Y238F^ restores MMR *in vitro*

To determine whether Y238 is biologically important in the context of MMR, we expressed HB-EV, HB-MSH2 and HB-MSH2^Y238F^ in HEK293 cells in which the expression of NPM-ALK is DOX-inducible.^4^ To assess MMR, cells were transiently transfected with the MMR β-galactosidase reporter plasmid (pCAR-OF)^4, 20^ This plasmid contains a 58-base-pair poly(C-A) tract at the 5′ end of its coding region, placing the start codon out of frame. Thus, in cells with dysfunctional MMR, MSI resulting from strand slippage at the C-A repeat can place the *β-galactosidase* gene in frame, resulting in measurable enzymatic activity.

**Figure 2:**
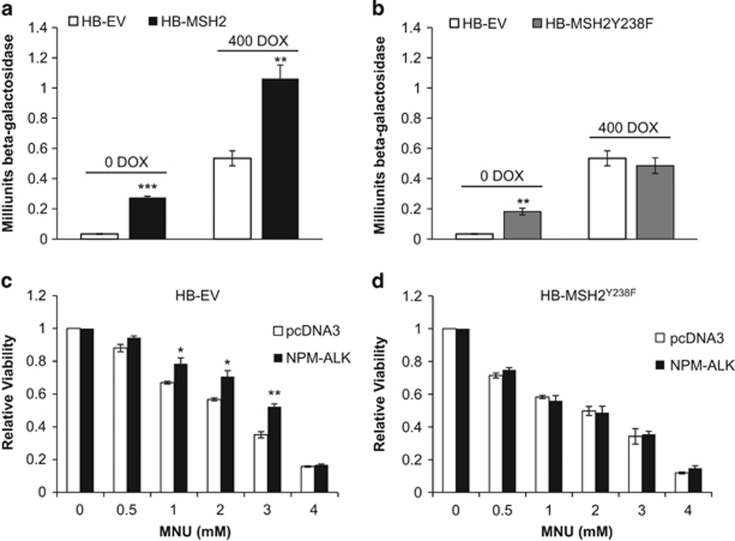
Enforced expression of HB-MSH2^Y238F^ restores MMR function *in vitro*. β-galactosidase MMR assay of Tet-on HEK293/NPM-ALK cells transfected with the pCAR-OF plasmid plus (**a**) HB-EV, HB-MSH2 and (**b**) HB-EV or HB-MSH2^Y238F^ for 24 h. The following day, the medium was supplemented with 0 ng/ml DOX (left) and 400 ng/ml DOX (right) to induce NPM-ALK expression. β-galactosidase activity, indicative of MMR dysfunction, was measured as outlined in the Materials and methods in triplicate 48 h post transfection, and at least three independent experiments were performed. Data are presented as mean milliunits of β-galactosidase±s.e.m. Statistical significance was measured by Student's *t*-test, where **P<0.01 and ***P<0.001. (**c**) GP293 cells transfected with HB-EV or (**d**) HB-MSH2^Y238F^ and pcDNA3 or NPM-ALK were treated with DMSO (0 mM), 0.5, 1, 2, 3, 4 or mM MNU for 48 h in 48-well plates. Viability was measured using the MTS cell viability; higher cell viability correlated with the loss of DNA MMR. Data are represented as mean relative cell viability (normalized to 0 mM and pcDNA3, which was set at 1)±s.e.m., and statistical significance was determined by Student's *t*-test, where *P<0.05 and **P<0.01. A representative result of three independent experiments is shown.

In HEK293 cells without NPM-ALK expression (that is, no DOX), enforced expression of HB-MSH2 or HB-MSH2^Y238F^ led to a statistically significant increase (*P*<0.001 and *P*<0.01) in β-galactosidase activity over cells transfected with HB-EV (Figures 2a and b). This suggests that overexpression of these MSH2 proteins alone can impair MMR, which substantiates previously published findings that overexpression of MMR proteins can lead to deregulation of MMR, mimicking what is seen in cells lacking certain MMR genes.^20, 23, 24^

With the induction of NPM-ALK (that is, 400 μg of DOX), we identified a significant increase in β-galactosidase, which is in concordance with our previous findings.^4^ Enforced expression of HB-MSH2 resulted in a further increase in β-galactosidase activity over cells expressing HB-EV (*P*<0.01) (Figure 2a, 400 DOX). In contrast, enforced expression of HB-MSH2^Y238F^ did not significantly alter the β-galactosidase activity compared with cells expressing HB-EV (Figure 2b, 400 DOX). These findings strongly suggest that MSH2^Y238F^ has a substantial biological difference from MSH2 in the presence of NPM-ALK.

To further assess whether enforced expression of HB-MSH2^Y238F^ has the ability to neutralize the MMR-deregulating effect of NPM-ALK, we transfected HB-EV or HB-MSH2^Y238F^ into GP293 cells with or without the co-transfection of NPM-ALK. These cells were subsequently treated with MNU to assess cell viability. Of note, MNU, a mono-functional methylating agent that induces aberrant bases, is widely used to assess MMR *in vitro.*^25^ MNU generates DNA-damage-induced apoptosis that is MMR-dependent. Thus, in the absence of MMR, cells are resistant to MNU treatment.^26^ As shown in Figure 2c, in GP293 cells transfected with HB-EV, cells co-transfected with NPM-ALK showed a significantly higher cell viability than those co-transfected with EV in the presence of 1–3 mM MNU (*P*<0.05, 1–2 mM; *P*<0.01, 3 mM). In contrast, in cells transfected with HB-MSH2^Y238F^, the co-transfection of NPM-ALK did not result in any significant difference in cell viability, as compared with those co-transfected with the EV (Figure 2d). Thus, the HB-MSH2^Y238F^ mutant appeared to have neutralized the MMR-deregulating effect of NPM-ALK.

### Enforced expression of HB-MSH2^Y238F^ alters MSH2:MSH6 (MutSα) binding

**Figure 3:**
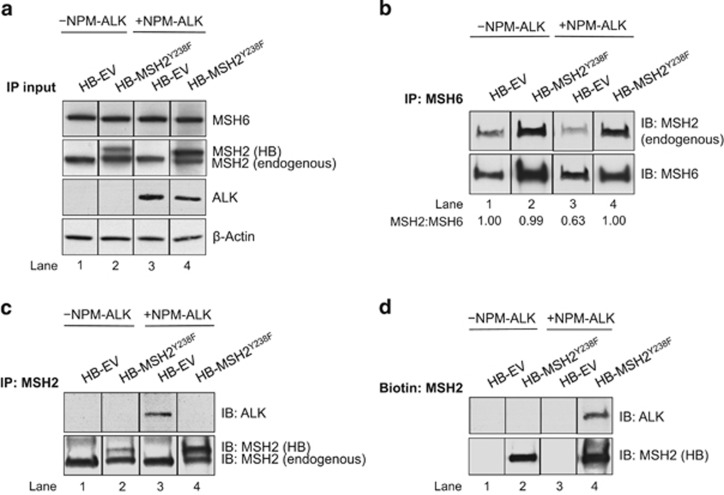
Enforced expression of HB-MSH2^Y238F^ alters MutSα (MSH2:MSH6) formation and the MSH2:NPM-ALK interaction. (**a**) Western blot analysis of GP293 cell lysate transfected with HB-EV or HB-MSH2^Y238F^ and pcDNA3 (lanes 1–2) or NPM-ALK (lanes 3–4). The western blot was probed with anti-MSH6 and anti-ALK antibodies. Anti-MSH2 antibody was used to detect endogenous MSH2 (lower band) and HB-MSH2/HB-MSH2^Y238F^ expression levels (upper band, HB). Anti-β-actin was used as a loading control. All panels are from the same western blot. (**b**) MSH6 was precipitated from cell lysate shown in **a** under co-IP conditions using an anti-MSH6 antibody. The resulting immunoblot was probed with MSH2 and MSH6 antibodies to measure the MSH2:MSH6 (MutSα) interaction. Densitometry shows the intensity of endogenous MSH2 normalized to MSH6, relative to lane 1. No exogenous MSH2 protein was detected by co-IP. All panels are from the same western blot. (**c**) MSH2 was precipitated from cell lysate **a** under co-IP conditions using an anti-MSH2 antibody, and the resulting immunoblot was probed with anti-ALK and anti-MSH2 to detect the interaction of MSH2 (endogenous and HB-tagged):NPM-ALK. All panels are from the same western blot. (**d**) Biotin purification of HB-MSH2^Y238F^ from **a**, probed with anti-ALK and anti-MSH2 to measure the HB-MSH2^Y238F^:NPM-ALK interaction. All panels are from the same western blot, and a representative result (from the same experiment) of three independent experiments is shown.

Previously published data from our group demonstrated that NPM-ALK expression blocked the interaction of MSH6 and MSH2,^4^ which is critical for MMR. We hypothesized that p-Y of MSH2^Y238^ is involved in this process. Thus, we performed coimmunoprecipitation experiments using GP293 cells transiently transfected with HB-EV or HB-MSH2^Y238F^. The cell lysate used for this experiment was initially subjected to western blotting, and the expression of endogenous and HB-tagged exogenous MSH2 proteins was confirmed (Figure 3a, lanes 1 and 2). Another set of cell lysates was similarly generated, except with the co-transfection of NPM-ALK (Figure 3a, lanes 3 and 4).

Using these two sets of cell lysates, the physical binding between MSH2 and MSH6 was assessed using coimmunoprecipitation with an anti-MSH6 antibody. In the context of MMR, MSH2 has multiple binding partners,^27, 28, 29^ whereas MSH6 only interacts with MSH2.^27^ Thus, using an anti-MSH6 antibody (rather than anti-MSH2) for the pull-down is more informative. As shown in Figure 3b, we only detected endogenous MSH2 bound to immunoprecipitated MSH6, suggesting that HB-MSH2^Y238F^ did not bind well to MSH6. Of note, in the absence of NPM-ALK (lane 1 and 2), when the variation in the amount of MSH6 protein pulled down was corrected, we found no difference between the interaction of MSH6 with MSH2, regardless of whether these cells were transfected with the EV or HB-MSH2^Y238F^. When NPM-ALK was co-transfected, there was a 40% reduction in the interaction between MSH6 and endogenous MSH2 (lane 3), and this finding is in agreement with our previous finding.^4^ Importantly, with enforced expression of HB-MSH2^Y238F^, the binding of MSH2 to MSH6 was almost completely restored (lane 4).

### Enforced expression of HB-MSH2^Y238F^ alters MSH2:NPM-ALK binding

Using the cell lysate depicted in Figure 3a, we assessed how MSH2^Y238F^ may affect the interaction of endogenous MSH2 with NPM-ALK. As shown in Figure 3c, when MSH2 was pulled down with an anti-MSH2 antibody, the interaction between NPM-ALK and endogenous MSH2 was readily detectable, as previously described.^4, 18^ In contrast, the MSH2:NPM-ALK interaction was not detectable by immunoprecipitation when HB-MSH2^Y238F^ was expressed (lane 4). When pull-down of HB-MSH2^Y238F^ was done by biotin pull-down assay, we readily identified an interaction between NPM-ALK and HB-MSH2^Y238F^ (Figure 3d, lane 4). Taken together, these findings suggest that HB-MSH2^Y238F^ blocks the interaction between NPM-ALK and endogenous MSH2, possibly by binding to NPM-ALK and sequestering it away from endogenous MSH2.

### Enforced expression of MSH2^Y238F^ decreases MSH2 p-Y and restores MSH2:MSH6 (MutSα) formation in ALK+ALCL cells

**Figure 4:**
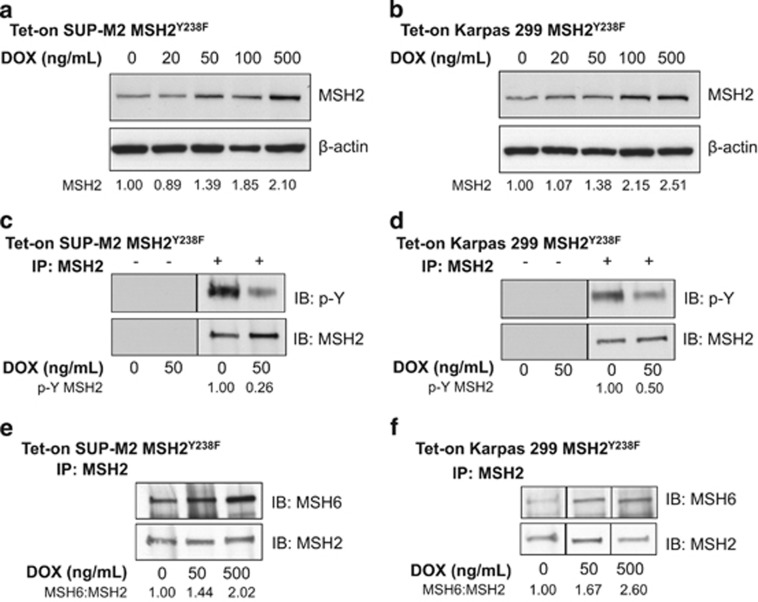
Enforced expression of MSH2^Y238F^ in ALK+ALCL cell lines alters MSH2 phosphorylation and MutSα formation. (**a**) Tet-on SUP-M2 MSH2^Y238F^ cell lysate, in which the expression of MSH2^Y238F^ correlated with the dose-dependent increase of DOX. (**b**) Tet-on Karpas 299 MSH2^Y238F^ cell lysate depicting the dose-dependent increase in MSH2^Y238F^ expression with DOX treatment. In **a** and **b**, a β-actin antibody was used as a loading control, and densitometry of MSH2 expression relative to β-actin is shown. (**c**) Tet-on SUP-M2 MSH2^Y238F^ and (**d**) Tet-on Karpas 299 MSH2^Y238F^ cells were treated with 0 and 50 ng/ml DOX, and the resulting cell lysate was subjected to IP using an anti-MSH2 antibody (+). p-Y of MSH2 was measured using an anti-p-Y antibody. No antibody controls (−) were included to account for nonspecific binding to the beads. Densitometry for phosphorylated MSH2 relative to total MSH2 is shown. (**e**) MSH2 was precipitated from Tet-on SUP-M2 MSH2^Y238F^ lysate and (**f**) Tet-on Karpas 299 MSH2^Y238F^ lysate treated with 0, 50 and 500 ng/ml DOX under co-IP conditions. The resulting immunoblots were probed with an anti-MSH6 and an anti-MSH2 antibody to assess the amount of MSH6 precipitated relative to MSH2. Densitometry for MSH6:MSH2 is shown. All panels in **f** are from the same western blot. Results are representative of at least three independent experiments.

The biological importance of MSH2^Y238^ phosphorylation in the context of NPM-ALK pathobiology was next validated in ALK+ALCL cell lines, which express NPM-ALK at the steady state. Using retroviral vectors, Karpas 299 and SUP-M2 cells stably transduced with an inducible MSH2^Y238F^ vector were generated, and were termed Tet-on MSH2^Y238F^ cells. The Tet-on SUP-M2 and Karpas 299 MSH2^Y238F^ cells showed a dose-dependent increase in the expression of total MSH2 protein upon DOX treatment (Figures 4a and b). Of note, both Karpas 299 and SUP-M2 have endogenous MSH2 expression, as evidenced at 0ng/ml DOX.

Using these Tet-on MSH2^Y238F^ cells, MSH2^Y238F^ expression was turned on at a low level (resulting in ∼40% increase in the total MSH2 protein level) and MSH2 phosphorylation was assessed by immunoprecipitation. The expression of MSH2^Y238F^ in SUP-M2 cells resulted in an ∼70% reduction in detectable p-Y (Figure 4c). Similar results were achieved in Karpas 299 cells, in which MSH2^Y238F^ decreased MSH2 phosphorylation by 50% (Figure 4d).

As the expression of MSH2^Y238F^ resulted in a decrease in detectable MSH2 p-Y, we then asked whether MSH2^Y238F^ was capable of restoring the interaction of MSH2:MSH6, which would indicate a partial restoration of MMR in these cells. As measured by co-IP using an anti-MSH2 antibody, expression of MSH2^Y238F^ in the Tet-on SUP-M2 cells (Figure 4e) and Karpas 299 cells (Figure 4f) resulted in a dose-dependent increase in the amount of MSH6 bound to MSH2.

### ALK+ALCL cells are more sensitive to DNA-damage-inducing drugs upon enforced expression of MSH2^Y238F^

**Figure 5:**
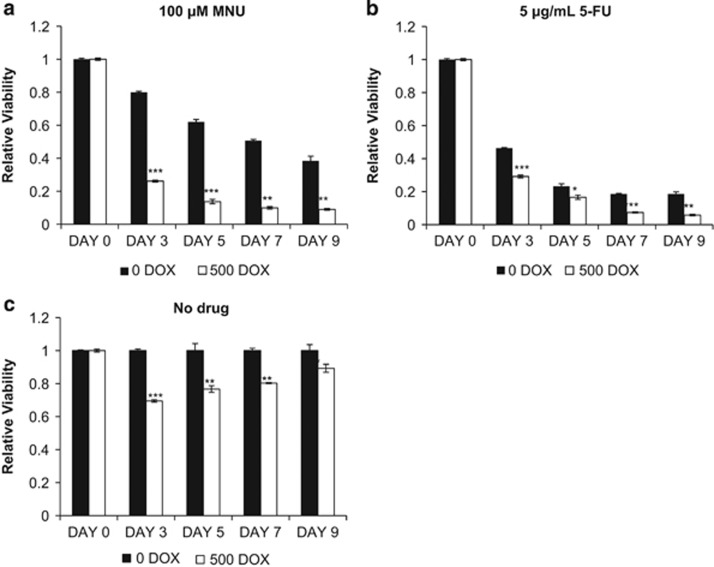
Enforced expression of MSH2^Y238F^ increases sensitivity to DNA-damage-inducing drugs in ALK+ALCL cells. Tet-on SUP-M2 MSH2^Y238F^ cells were plated in six-well plates and treated with 0 ng/ml DOX and 500 ng/ml DOX, followed by the addition of (**a**) 100 μM MNU, (**b**) 5 μg/ml 5-FU or (**c**) DMSO for 24 h. Cells were then plated in 48-well plates (day 0) and viability was assessed by MTS assay every 2 days until day 9. Viability is displayed relative to DMSO/0 DOX, which was set at one. Statistical significance was calculated by Student's *t*-test where *P<0.05, **P<0.01 and ***P<0.001. All samples were measured in sextuplicate, and a representative result of three independent experiments is shown.

As our previous results have shown that MSH2^Y238F^ is capable of restoring MSH2:MSH6 formation in ALK+ALCL, we then asked whether cells expressing MSH2^Y238F^ also have restored MMR function. Owing to technical reasons, we were unable to use the pCAR-OF plasmid described above, as it would have involved multiple gene transfection into ALK+ALCL cells, which significantly decreased cell viability (not shown). Instead, we assessed MMR indirectly by treating these cells with MNU. In addition, we also assessed MMR by treating these cells with the thymidylate synthase inhibitor 5-FU, as resistance to 5-FU has been associated with loss of MMR.^30^ As shown in Figures 5a and b, experiments using both MNU and 5-FU showed similar results. The expression of MSH2^Y238F^ (induced by the addition of 500 ng/ml of DOX) in the presence of the DNA-damaging drugs resulted in a significant increase in loss of viability from day 3 onward. These findings support the concept that MSH2^Y238F^ restores MMR in ALK+ALCL cells. Interestingly, expression of MSH2^Y238F^ alone, without the addition of MNU or 5-FU, resulted in a significant loss of viability (Figures 5c, *P*<0.001 until day 7). The decrease in statistical significance on day 9 can be attributed to the fact that only one dose of DOX was given at the beginning of the experiment, and the expression of MSH2^Y238F^ slowly faded away.

### Enforced expression of MSH2^Y238F^ induces spontaneous apoptosis in ALK+ALCL cells

As our results depicted in Figure 5 suggest that expression of MSH2^Y238F^ alone can decrease cell viability of ALK+ALCL cells, we performed studies to analyze changes in the cell cycle and apoptosis induced by MSH2^Y238F^. By propidium iodide staining, at 48 and 120 h after MSH2^Y238F^ expression, there was a significant increase in the fraction of apoptotic cells in the Tet-on SUP-M2 MSH2^Y238F^ cell line (Supplementary Figure 3). The average percentage of cells in each stage of cell cycle is shown in Supplementary Table 2 (*n*=3). At 48 and 120 h post MSH2^Y238F^ expression, the increase in the percentage of cells in the apoptotic/SubG1 phase was statistically significant (*P<*0.01).

**Figure 6:**
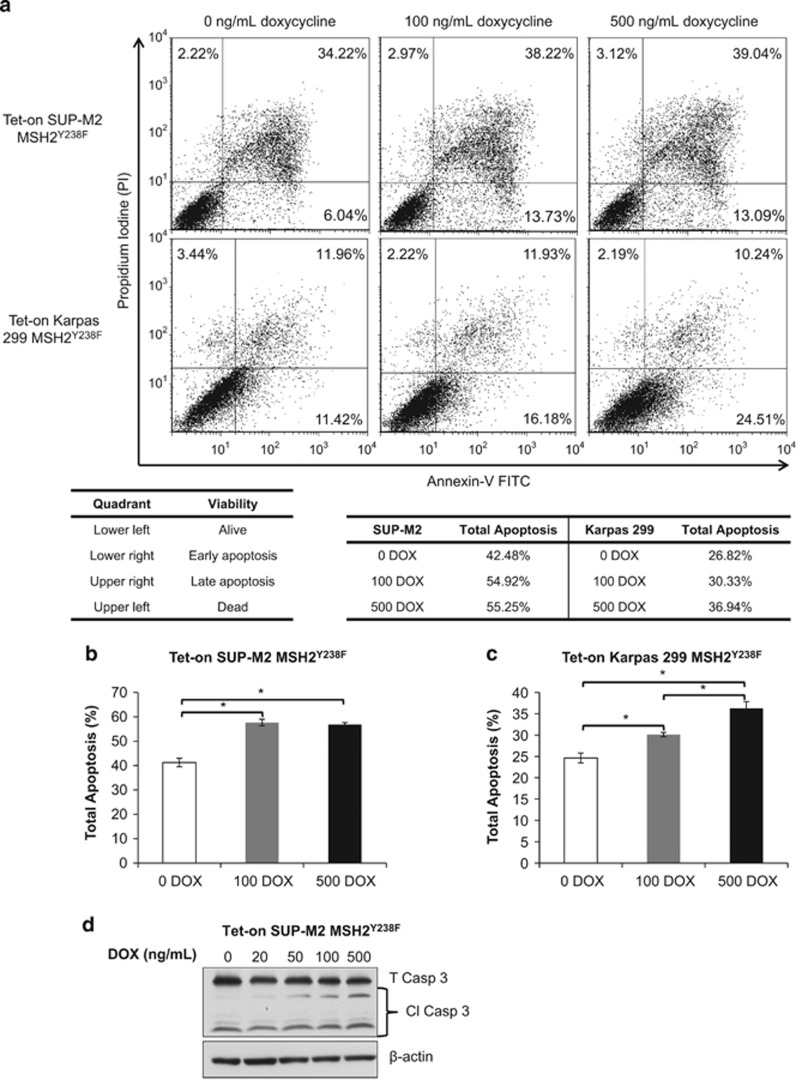
Enforced expression of MSH2^Y238F^ induces spontaneous apoptosis in ALK+ALCL cells. (**a**) Tet-on SUP-M2 MSH2^Y238F^ (upper three panels) and Tet-on Karpas 299 MSH2^Y238F^ (lower three panels) were treated with 0, 100 and 500 ng/ml DOX to enforce MSH2^Y238F^ expression, were stained with Annexin-V/FITC/propidium iodide and were subjected to flow cytometry analysis for apoptosis. The lower left table shows the distribution of the cells in the different stages of apoptosis. The lower right table summarizes the total percent apoptosis (early, late and dead) for each treatment group. The experiment was repeated three times, and a representative result is shown. (**b**) The percentage of Tet-on SUP-M2 MSH2^Y238F^ and (**c**) Tet-on Karpas 299 MSH2^Y238F^ cells in all apoptotic stages (early, late and dead) was calculated from three independent experiments, and the mean percent total apoptosis±s.e.m. is shown. Statistical significance was determined by Student's *t*-test, where *P<0.05. (**d**) Western blot analysis of the apoptotic marker cleaved caspase 3 in the Tet-on SUP-M2 MSH2^Y238F^. The immunoblot was probed with anti-caspase 3 to detect total (T) and cleaved (Cl) caspase 3 expression. Anti-β actin was used as a loading control. Representative results from two independent experiments are shown.

Tet-on SUP-M2 and Karpas 299 MSH2^Y238F^ cells were subjected to Annexin V/propidium iodide staining to determine whether the expression of MSH2^Y238F^ resulted in a change in cells undergoing early- or late-stage apoptosis. As shown in Figure 6a, increasing expression of MSH2^Y238F^ resulted in a dose-dependent increase in the fraction of cells undergoing both early (lower right quadrant) and late (upper right quadrant) apoptosis.

A graphical representation of the total apoptotic Tet-on SUP-M2 MSH2^Y238F^ cells is shown in Figure 6b (*n*=3); when the percentages of early, late and dead cells were added together (that is, total apoptotic cells), a significant increase in apoptosis was measured in cells treated with 100 ng/ml DOX (*P*<0.05). In the Tet-on Karpas 299 MSH2^Y238F^ cells, a significant dose-dependent increase in the percentage of total apoptotic cells was detectable in cells treated with both 100 and 500 ng/ml DOX (*P*<0.05) (Figure 6c). Apoptosis in the Tet-on SUP-M2 MSH2^Y238F^ cells was confirmed by western blotting for cleaved caspase 3 expression (Figure 6d).

## Discussion

The finding that oncogenic tyrosine kinases can impair MMR is relatively recent, and has not been extensively studied. In 2008, it was described that BCR-ABL, an oncogenic fusion tyrosine kinase, can induce MSI, but the underlying mechanism was not investigated.^31^ In a previous publication from our laboratory, we found that the oncogenic tyrosine kinase NPM-ALK induced the phosphorylation of MSH2 at unknown tyrosine residues, leading to MMR dysfunction. We also reported that ALK+ALCL cell lines and patient samples exhibited MSI and abnormal MSH2 cytoplasmic localization, which are both evidence of MMR deficiency.^4^ In this study, we tested the hypothesis that p-Y of MSH2 is a critical step in the deregulation of MMR by NPM-ALK. First, using liquid chromatography–mass spectrometry, we report for the first time that Y238 is a site that can be tyrosine phosphorylated by NPM-ALK. Second, we created the MSH2^Y238F^ mutant and collected evidence that Y238 is the predominant site of NPM-ALK-mediated phosphorylation. Third, by performing various functional assays using MSH2^Y238F^ in GP293 and ALK+ALCL cells, we found that MSH2^Y238F^ can restore MMR. These findings support the concept that p-Y of MSH2^Y238^ is a critical step by which NPM-ALK deregulates MMR.

**Figure 7:**
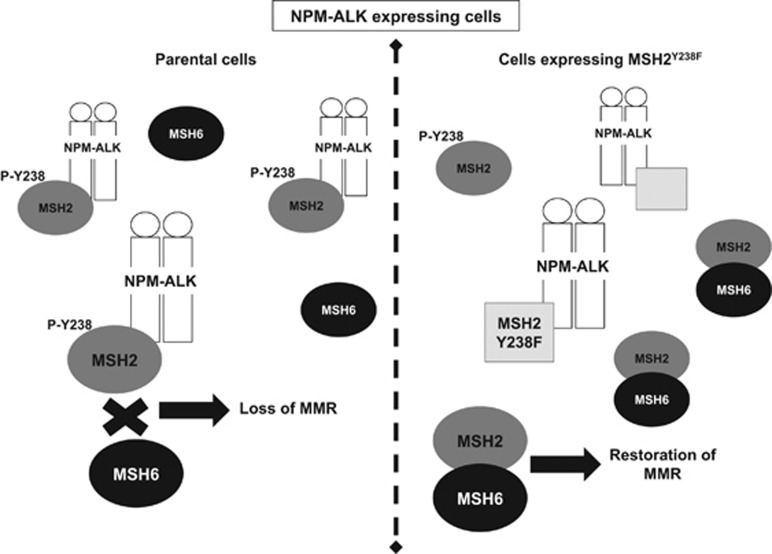
Schematic model of how MSH2^Y238F^ is postulated to restore MMR. Our working model based on data published in this manuscript, as well as our previously reported findings,^4^ is that NPM-ALK, through an interaction with MSH2 that leads to MSH2 p-Y at residue 238, sequesters MSH2 from MSH6, blocking MutSα formation and its translocation to the nucleus in the presence of DNA damage. Our data from this manuscript, using both GP293 and ALK+ALCL cells, suggest that when its expression is enforced MSH2^Y238F^ blocks the phosphorylation of *endogenous* MSH2, by preferentially binding to NPM-ALK. Endogenous MSH2 is then capable of binding to MSH6, forming the MutSα heterodimer, and restoring MMR, even in the presence of NPM-ALK.

One of the key findings of this study is that enforced expression of MSH2^Y238F^ in the presence of NPM-ALK restores MMR using both GP293 and ALK+ALCL cell lines. The *β-galactosidase* reporter assay and the MNU sensitivity assay have been used previously to assess the MMR function in other experimental systems.^4, 20, 32, 33^ Although the mechanism by which MSH2^Y238F^ restores MMR needs further delineation, we believe that results from this study have provided important clues. First, we found that the MSH2:MSH6 binding was disrupted in the presence of NPM-ALK,^4^ and this defect was restored by MSH2^Y238F^. Second, in the presence of NPM-ALK, the p-Y of MSH2 decreased markedly after MSH2^Y238F^ expression. Third, the expression of MSH2^Y238F^ substantially reduced the binding between NPM-ALK and endogenous MSH2, although the mutant itself can bind to NPM-ALK. Taken together, the most likely scenario we have considered is that MSH2^Y238F^ restores MMR by sequestering NPM-ALK away from the endogenous MSH2 protein, leading to a substantial increase in the availability of endogenous MSH2:MSH6 heterodimers. This is demonstrated in a model shown in Figure 7, consistent with the observation that MSH2^Y238F^ exerts a dominant negative effect on NPM-ALK-mediated p-Y of endogenous MSH2.

The finding of phosphorylation of MSH2 at tyrosine 238 directly affecting its MMR function is highly novel. Y238 is located in the connector domain of the MSH2 protein, which allows for the intramolecular interactions of MutSα with downstream proteins.^34^ In the context of MMR function, a number of clinically relevant missense mutations have been identified in the connector domain of the MSH2 protein in Lynch Syndrome patients, leading to loss of MMR and MSH2 protein expression through a decrease in MSH2 protein stability.^35, 36^ Thus, phosphorylation of the connector domain of the MSH2 protein could significantly affect its ability to bind downstream proteins required to initiate the MMR signaling cascade.

To further understand the significance of p-Y of MSH2, we assessed the effect of the MSH2^Y238F^ mutant on the sensitivity to DNA-damaging agents in ALK+ALCL cells, specifically MNU and 5-FU, which induce DNA damage processed directly and indirectly by MMR. Cells with MMR deficiencies have been reported to be more resistant to these DNA-damaging drugs.^30, 37, 38, 39, 40, 41, 42, 43^ Thus, a restoration of sensitivity is associated with the return of MMR. In the context of MMR, MNU treatment creates *O*^*6*^-methylguanine lesions in the DNA that are mispaired with thymine (T), and the resulting *O*^*6*^-methylguanine:T mismatch is detected by MMR proteins. Through the consequent repair process, a T is reinserted opposite the *O*^*6*^-methylguanine, reinitiating a futile MMR cycle that results in double-stranded DNA breaks, G2 arrest and cell death.^26^ MMR processing after 5-FU treatment is similar to that seen with MNU; 5-FU can cause dNTP pool imbalances, leading to frequent 5-FU:Guanine lesions detected by MutSα, leading to MMR-dependent activation of the G2 checkpoint and apoptosis.^30^ We provide evidence that the expression of the MSH2^Y238F^ mutant significantly increased the sensitivity to MNU and 5-FU, suggesting a restoration of MMR in ALK+ALCL cells.

Our data also suggest that enforced expression of MSH2^Y238F^ alone can induce apoptosis in two ALK+ALCL cell lines. Exactly how this mutant mediates this biological effect is unknown. Nevertheless, apart from its role in MMR, MSH2 is known to carry important roles in the regulation of cell cycle checkpoint and apoptosis in both MMR-proficient and -deficient cells.^44^ It has been reported that overexpression of MSH2 induced cell death in MMR-functional GP293 and MMR-deficient SK-UT-1 cells.^17^ Loss of MMR has been associated with a decrease in apoptosis and an increase in UVB-associated DNA-damage adducts in the epidermis of *MSH2*-null mice, accompanied by an increase in UVB-associated tumors and loss of phosphorylated p53.^45, 46, 47^ The resistance of MMR-deficient cells to certain DNA-damaging agents is partially through a loss of DNA-damage-induced cell cycle arrest; MSH2 associates with the checkpoint proteins ATR, CHK1 and CHK2, both *in vitro* and *in vivo*, mediating the initiation of G2/M cell cycle arrest.^40, 48, 49^ Although we have not yet understood the exact mechanisms underlying the induction of apoptosis following overexpression of MSH2^Y238F^, we can speculate that ALK+ALCL cell lines carry a large number of DNA-damage adducts that, upon restoration of MMR, lead to an MMR-driven apoptotic signaling cascade.

There is accumulating evidence that cancers with oncogenic tyrosine kinases have dysfunctional DNA-damage responses directly related to kinase-driven aberrant downstream signaling processes.^50, 51, 52^ As another oncogenic tyrosine kinase, BCR-ABL, has been shown to inhibit MMR,^31^ and receptor tyrosine kinases have similar features in regard to activity and signal transduction, MMR dysfunction through MSH2 phosphorylation may be shared across multiple oncogenic tyrosine kinases. Furthermore, with the emergence of ALK as a molecular driver of a number of other cancer types, including non-small-cell lung cancer, diffuse large B-cell lymphoma, breast cancer, retinoblastoma, colon carcinoma and esophageal squamous cell carcinoma,^53^ we believe that detection of MSH2 phosphorylation at Y238 may be of great clinical and diagnostic significance. We believe that p-Y of MSH2 at Y238 and the subsequent inhibition of DNA MMR may be a universal mechanism by which oncogenic tyrosine kinases potentiate tumorigenesis. We are currently in the process of characterizing a phospho-MSH2^Y238^-specific antibody in our laboratory, and we are assessing its utility for clinical use (manuscript in preparation).

In conclusion, we would like to reiterate our findings that overexpression of wild-type MSH2 (in the form of HB-MSH2) consistently resulted in suppression, rather than improvement, of the MMR function. Although we were initially perplexed with these seemingly paradoxical results, our literature search has revealed that multiple overexpression of key DNA MMR genes (for example, MSH2) suppresses MMR.^20, 23, 24, 54, 55^ Although the mechanism underlying this paradoxical effect is not completely understood, we speculate that this may be related to the fact that DNA MMR proteins (for example, MSH2) often require heterodimerization with another partner, such as MSH6. Thus, it is perceivable that gene transfection of wild-type MSH2 may substantially decrease the formation of the MSH2:MSH6 heterodimer relative to other MSH2 heterodimers (with partners other than MSH6), which is suboptimal for MMR. The observation that overexpression of the MSH2^Y238F^ mutant, but not wild-type MSH2, can result in improvement of MMR also highlights the concept that the biological effects associated with this MSH2 mutant are not simply due to the expression of an excessive amount of MSH2 protein.

## Conclusion

In summary, we have presented evidence that NPM-ALK phosphorylates MSH2 at a specific site, Y238, creating a functional loss of MMR. Our data suggest that enforced expression of the MSH2^Y238F^ phosphorylation-less mutant is able to restore MMR through a dominant-negative effect, whereby its expression blocks NPM-ALK interaction with endogenous MSH2, allowing for the formation of the endogenous MutSα heterodimer. The importance of detecting MSH2 p-Y in oncogenic tyrosine kinase-expressing cancers clinically needs to be further defined.
