# Supplementary material for: NPM-ALK mediates phosphorylation of MSH2 at tyrosine 238, creating a functional deficiency in MSH2 and the loss of mismatch repair
Source: Blood Cancer J. 2015 May 15;5(5):e311–. doi: 10.1038/bcj.2015.35 (PMC4476014; doi:10.1038/bcj.2015.35)
Supplement: Supplementary Information [file bcj201535x1.docx]

**NPM-ALK mediates phosphorylation of MSH2 at tyrosine 238, creating a functional deficiency in MSH2 and the loss of mismatch repair**

**Supplemental Files**

**Supplemental Methods**

**Generation of Tet-on ALK+ALCL MSH2/MSH2^Y238F^ cell lines**

Tet-on ALK+ALCL MSH2^Y238F^ cell lines were generated using the RetroX Tet-on Advanced Inducible Expression System (Clontech, Mountain View, CA). To generate the viral supernatant, Phoenix packaging cells were transfected with the pRetro-X Tet-on Advanced plasmid using Lipofectamine 2000. The cell supernatant was collected 48 hours post-transfection, filtered and was used to resuspend 5 X 10^6^ Karpas 299 and SUP-M2 cells, along with 6 μg/mL polybrene (Santa Cruz Biotechnology, Santa Cruz, CA). The cells were placed in a 25 cm^2^ flask and were centrifuged 1.5 hours at 2000 X g, 32° Celsius, after which fresh media was added to the cells. The following day, the cells were reinfected. The cells were washed three times 24 hours later and plated in media with 200 μg/mL G418,100 units/mL penicillin and 100 μg/mL streptomycin to select for stable cells.

MSH2^Y238F^ cDNA was cloned by Genscript (Piscataway, NJ) into the pRetroX Tight-Pur vector (Clontech). To generate the double stable RetroX Tet-on Karpas 299 and SUP-M2 MSH2^Y238F^ Advanced cell lines, GP2-293 cells were transfected with the pRetroX Tight-Pur MSH2 or MSH2^Y238F^ vector along with the pAmpho vector (Clontech) using Lipofectamine 2000. Forty-eight hours post-transfection, 5 X 10^6^ Tet-on SUP-M2 and Karpas 299 cells were resuspended in viral supernatant. Following retroviral infection, the cells were grown in RPM1-1640 media with 10% Tet-System Approved FBS (Clontech), 200 μg/mL G418, 100 units/mL penicillin,100 μg/mL streptomycin and 2.2 μg/mL puromycin (Invitrogen).

**Supplemental Figures**

1

934

DNA binding domain 1-124

Connector domain 125-299

Core domain 300-488 and 554-619

Clamp domain 489-553

ATPase domain 620-820

Helix-turn-helix domain 820-924

MSH6 and MSH3 interaction sites 378-625 and 875-934

*

**Supplemental Figure 1. Predicted tyrosine phosphorylation sites in the MSH2 protein.** MSH2 has 13 predicted tyrosine phosphorylation residues**.** Schematic diagram showing predicted MSH2 tyrosine phosphorylation residues in the MSH2 protein by domain, as proposed by NetPhos 2.0.[^1^](#_ENREF_1) The highest predicted tyrosine phosphorylation site was at Y238 (phosphorylation score 0.980; denoted by * in the Figure), located in the MSH2 connecter domain. Figure adapted from Ollila *et al*., 2006.[^2^](#_ENREF_2)


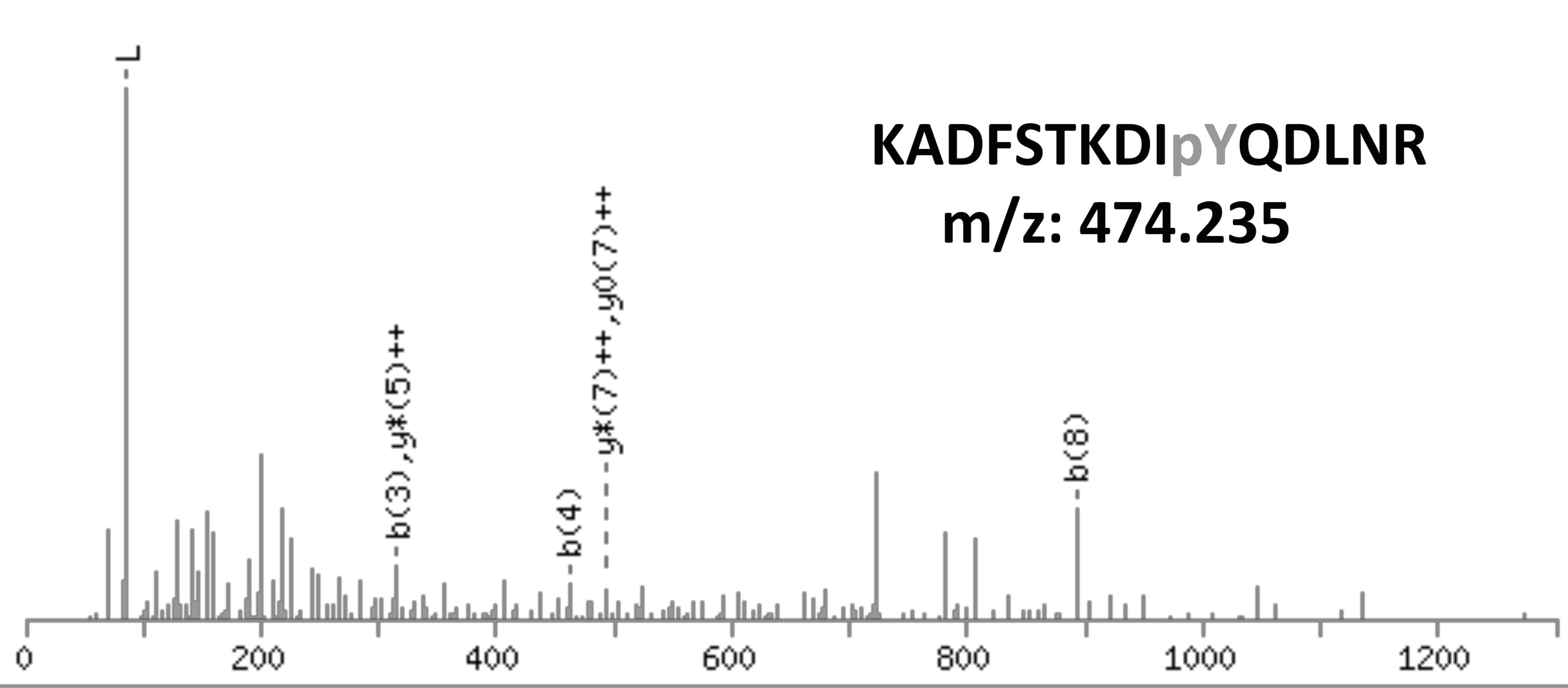


**Supplemental Figure 2**. **MSH2 is tyrosine phosphorylated by NPM-ALK at residue 238 by mass spectrometry.** The tandem affinity mass spectrometry (MS/MS) spectrum of a representative MSH2 peptide showing Y238 phosphorylation. m/z: peptide mass to charge ratio.

**
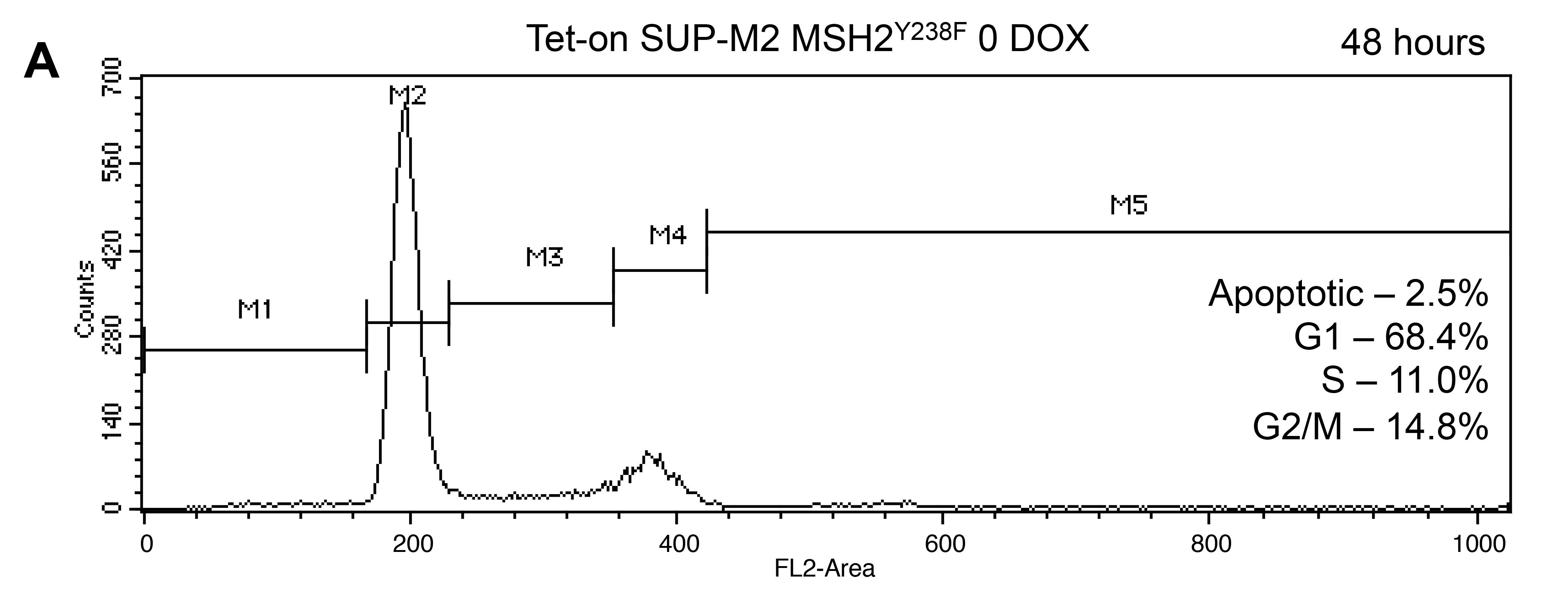
**

**
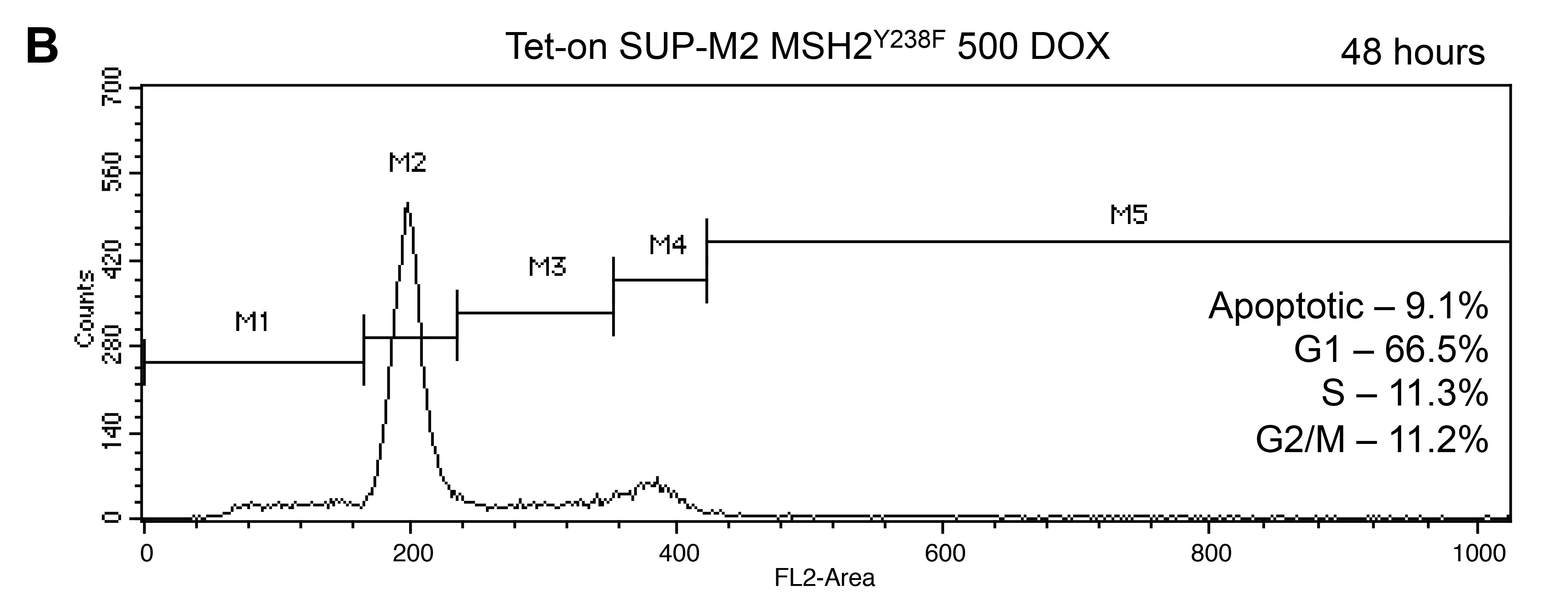
**

**
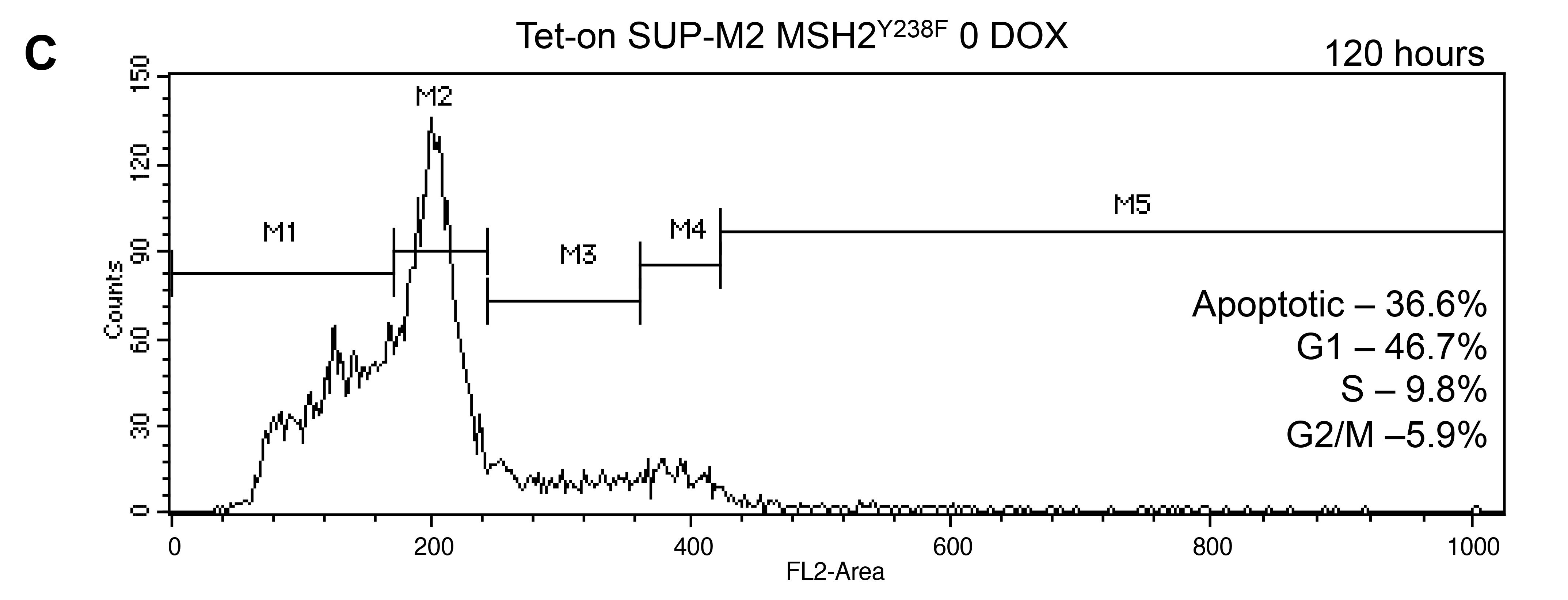
**

**
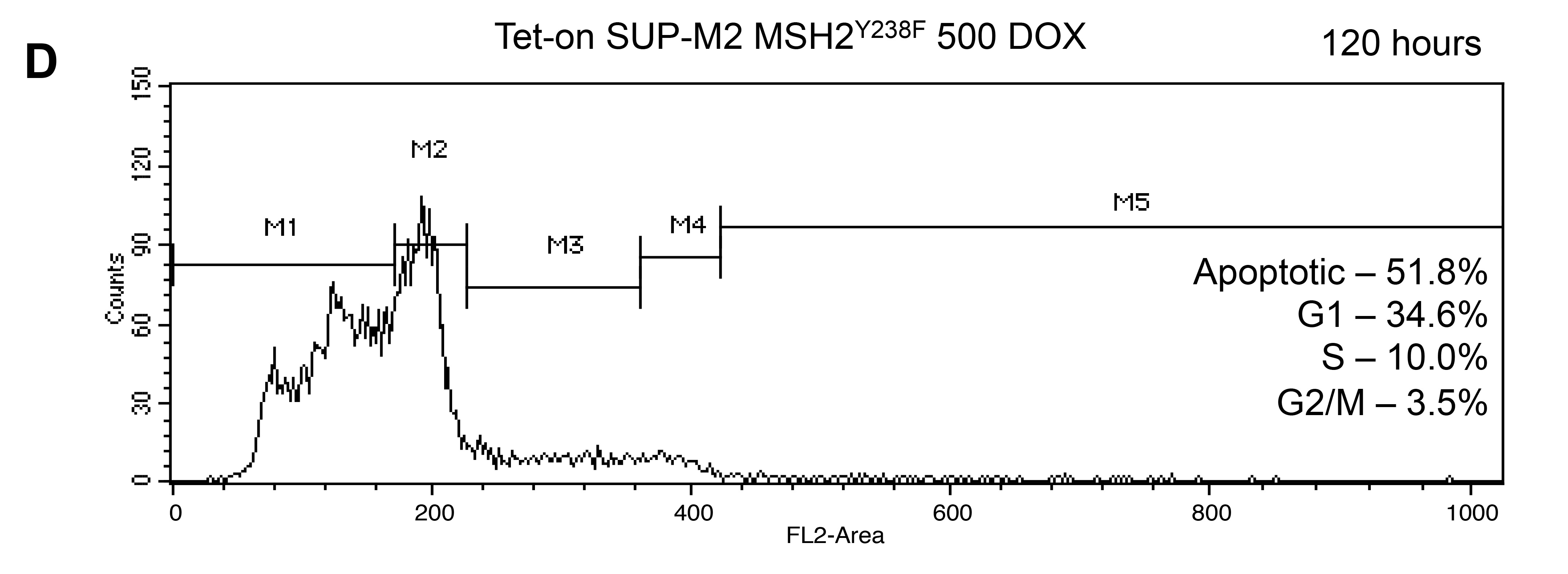
**

**Supplemental Figure 3**. **Enforced expression of MSH2^Y238F^ shifts cells into the SubG1 phase/apoptotic phase.** (A) Tet-on SUP-M2 cells treated with 0 ng/mL and (B) 500 ng/mL doxycycline for 48 and (C) 0 ng/mL and (D) 500 ng/mL doxycycline for 120 hours were stained with propidium iodide and analyzed by flow cytometry for cell cycle distribution. A representative result of 3 independent experiments is shown.

**Supplemental Tables**

**Supplemental Table 1.** NetPhos 2.0^a^ server predicted tyrosine phosphorylation sites in the MSH2 protein sequence.

| **Amino Acid Sequence** | **Position** | **Phosphorylation Score** |
| --- | --- | --- |
| RGDF*YTAHG | 43 | 0.863 |
| RVEV*YKNRA | 103 | 0.815 |
| ENDW*YLAYK | 118 | 0.867 |
| VGVG*YVDSI | 165 | 0.657 |
| TKDI*YQDLN | 238 | 0.981 |
| LNEE*YTKNK | 563 | 0.896 |
| NKTE*YEEAQ | 570 | 0.974 |
| ISSG*YVEPM | 588 | 0.768 |
| APVP*YVRPA | 619 | 0.837 |
| PNDV*YFEKD | 656 | 0.908 |
| GKST*YIRQT | 678 | 0.556 |
| EEFQ*YIGES | 856 | 0.830 |
| ESQG*YDIME | 863 | 0.910 |

^a^Online prediction software[^1^](#_ENREF_1)

*Predicted phosphorylation site

**Supplemental Table 2.** Cell cycle analysis of Tet-on SUP-M2 MSH2^Y238F^ cells after enforced expression of MSH2^Y238F^.

| **Cell Cycle (48 hours)** | **0 DOX (± SEM)** | **500 DOX (± SEM)** | ***P* value** |
| --- | --- | --- | --- |
| SubG1 | 4.0 ± 1.1 | 9.5 ± 0.3 | ** |
| G1 | 67.2 ± 2.0 | 62.7 ± 4.2 | ns |
| S | 8.8 ± 1.8 | 9.6 ± 1.5 | ns |
| G2/M | 12.8 ± 1.4 | 9.7 ± 0.9 | ns |
| **Cell Cycle (120 hours)** | **0 DOX (± SEM)** | **500 DOX (± SEM)** | ***P* value** |
| SubG1 | 31.4 ± 2.9 | 51.2 ± 0.7 | ** |
| G1 | 49.0 ± 1.7 | 32.6 ± 1.3 | ** |
| S | 9.9 ± 0.7 | 9.9 ± 0.1 | ns |
| G2/M | 6.2 ± 0.2 | 3.8 ± 0.1 | *** |

SEM – standard error of the mean

DOX – doxycycline

ns – non-significant, ***P*<0.01, ****P*<0.001 (Student’s T-test)

**Supplemental References**

1. Blom N, Gammeltoft S, Brunak S. Sequence and structure-based prediction of eukaryotic protein phosphorylation sites. *J Mol Biol* 1999;**294**(5):1351-1362.

2. Ollila S, Sarantaus L, Kariola R, et al. Pathogenicity of MSH2 missense mutations is typically associated with impaired repair capability of the mutated protein. *Gastroenterology* 2006;**131**(5):1408-1417.
